# Ischemic Benefit and Hemorrhage Risk of Ticagrelor-Aspirin Versus Aspirin in Patients With Acute Ischemic Stroke or Transient Ischemic Attack

**DOI:** 10.1161/STROKEAHA.121.035555

**Published:** 2021-09-30

**Authors:** S. Claiborne Johnston, Pierre Amarenco, Maria Aunes, Hans Denison, Scott R. Evans, Anders Himmelmann, Marianne Jahreskog, Stefan James, Mikael Knutsson, Per Ladenvall, Carlos A. Molina, Sven Nylander, Joachim Röther, Yongjun Wang

**Affiliations:** Dean’s Office, Dell Medical School, University of Texas at Austin (S.C.J.).; Department of Neurology and Stroke Center, Bichat Hospital, Paris University, France (P.A.).; AstraZeneca, Biopharmaceuticals R&D, Gothenburg, Sweden (M.A., H.D., A.H., M.J., M.K., P.L., S.N.).; Biostatistics Center, George Washington University (S.R.E.).; Department of Medical Sciences, Uppsala University, Sweden (S.J.).; Stroke Unit, Hospital Vall d’Hebron, Barcelona, Spain (C.A.M.).; Department of Neurology, Asklepios Klinik Altona, Hamburg, Germany (J.R.).; Department of Neurology, Tiantan Hospital, Capital Medical University, Beijing, China (Y.W.).

**Keywords:** aspirin, benefit-risk assessment, ischemic attack, transient, ischemic stroke, ticagrelor, treatment outcome

## Abstract

**Background and Purpose:**

In patients with acute mild-moderate ischemic stroke or high-risk transient ischemic attack, the THALES trial (Acute Stroke or Transient Ischemic Attack Treated With Ticagrelor and Aspirin for Prevention of Stroke and Death) demonstrated that when added to aspirin, ticagrelor reduced stroke or death but increased risk of severe hemorrhage compared with placebo. The primary efficacy outcome of THALES included hemorrhagic stroke and death, events also counted in the primary safety outcome. We sought to disentangle risk and benefit, assess their relative impact, and attempt to identify subgroups with disproportionate risk or benefit.

**Methods:**

In a randomized, placebo-controlled, double-blind trial of patients with mild-to-moderate acute noncardioembolic ischemic stroke or high-risk transient ischemic attack, patients were randomized within 24 hours after symptom onset to a 30-day regimen of either ticagrelor plus aspirin or matching placebo plus aspirin. For the present analyses, we defined the efficacy outcome, major ischemic events, as the composite of ischemic stroke or nonhemorrhagic death, and defined the safety outcome, major hemorrhage, as intracranial hemorrhage or hemorrhagic death. Net clinical impact was defined as the combination of these 2 end points.

**Results:**

In 11 016 patients (5523 ticagrelor-aspirin and 5493 aspirin), a major ischemic event occurred in 294 patients (5.3%) in the ticagrelor-aspirin group and in 359 patients (6.5%) in the aspirin group (absolute risk reduction 1.19% [95% CI, 0.31%–2.07%]). Major hemorrhage occurred in 22 patients (0.4%) in the ticagrelor-aspirin group and 6 patients (0.1%) in the aspirin group (absolute risk increase 0.29% [95% CI, 0.10%–0.48%]). Net clinical impact favored ticagrelor-aspirin (absolute risk reduction 0.97% [95% CI, 0.08%–1.87%]). Findings were similar when different thresholds for disability were applied and over a range of predefined subgroups.

**Conclusions:**

In patients with mild-moderate ischemic stroke or high-risk transient ischemic attack, ischemic benefits of 30-day treatment with ticagrelor-aspirin outweigh risks of hemorrhage.

**Registration:**

URL: https://www.clinicaltrials.gov; Unique identifier: NCT03354429.

## Abstract 2

Supplemental Digital Content is available in the text.

Antiplatelet agents and anticoagulants have been shown to reduce ischemic events in a variety of clinical settings, including in specific populations of patients with ischemic stroke or transient ischemic attack (TIA).^1,2^ This benefit is accompanied by an increase in risk of hemorrhage. Primary efficacy outcomes frequently include safety elements, such as hemorrhagic events, often obscuring an understanding of benefit in relationship to risk since individual events are included in both benefit and safety end points. The problem is exacerbated when outcomes are compared across different trials. While a single composite outcome measure that incorporates risks and benefits could be utilized to avoid confusion in weighing risks and benefits, such composites obscure the actual tradeoffs of treatments that inherently carry risks.

The THALES trial (Acute Stroke or Transient Ischemic Attack Treated With Ticagrelor and Aspirin for Prevention of Stroke and Death) demonstrates this issue.^3^ In 11 016 patients with acute mild-to-moderate ischemic stroke or TIA randomized to 30-day treatment with ticagrelor combined with aspirin (ticagrelor-aspirin) or aspirin alone, ticagrelor-aspirin reduced stroke or death (primary efficacy outcome) but, to a lesser degree in absolute terms, increased severe hemorrhage (primary safety outcome). Its primary efficacy outcome, stroke, or death included hemorrhagic events that were also counted in the primary safety outcome. Thus, weighing these outcomes is an inaccurate assessment of risk and benefit and obscures detection of subgroups that may experience disproportionate benefit or harm. Furthermore, the original publication of THALES results focused on relative risks whereas absolute risks provide a more useful comparison of risks and benefits.

To more clearly assess the benefits and risks of treatment with ticagrelor-aspirin, we redefined efficacy and safety outcomes to be exclusive and associated with similar long-term impact and reanalyzed results of the THALES trial. We evaluated presenting characteristics, demographics, and other factors associated with disproportionate absolute risk and benefit.

## Methods

### Data Sharing Statement

Data underlying the findings described in this article may be obtained in accordance with AstraZeneca’s data sharing policy described at: https://astrazenecagrouptrials.pharmacm.com/ST/Submission/Disclosure.

### Trial Design and Oversight

THALES was a randomized, double-blind, placebo-controlled, multicenter, international, parallel-group trial (NCT03354429) conducted at 414 sites in 28 countries.^3^ The Executive Committee designed and oversaw the conduct of the trial in collaboration with the sponsor, AstraZeneca. An independent Data Monitoring Committee regularly oversaw the safety of the patients and the integrity and conduct of the study throughout the trial. Details of the study rationale, design, methods, and study assumptions have been described previously.^3,4^

The trial was approved by the relevant ethics committee for each participating site. The trial analyses were performed by the sponsor under the direction of the Executive Committee. The first author had full access to the data and wrote the first draft of the article. The article was reviewed, edited, and approved by all authors, who decided to publish the data. The authors vouch for the accuracy and completeness of the data. The data reported here are in alignment with the CONSORT reporting requirements.^5^

### Study Population

Eligible patients randomized in THALES were 40 years of age or above, had a noncardioembolic acute ischemic stroke with a National Institutes of Health Stroke Scale score (range 0–42, higher scores indicate more severe stroke) of ≤5 or high-risk TIA (ABCD^2^ stroke risk score [scores assessing the risk of stroke on the basis of age, blood pressure, clinical features, duration of transient ischemic attack, and presence or absence of diabetes; range 0 (lowest risk) to 7 (highest risk)] of ≥6)^6^ or symptomatic intracranial or extracranial stenosis (≥50% narrowing in the diameter of the lumen of an artery that could account for the TIA). Randomization was required to occur within 24 hours after onset of symptoms. Before randomization, patients had undergone a computed tomography or magnetic resonance imaging scan of the brain to rule out intracranial hemorrhage (ICH) or other pathology that could explain the symptoms or contraindicate study treatment. Patients were not eligible if there was a history of atrial fibrillation, ventricular aneurysm, or suspicion of cardioembolic cause for the index TIA or stroke; planned carotid endarterectomy that required halting study medication within 3 days of randomization; known bleeding diathesis or coagulation disorder; history of previous symptomatic nontraumatic intracerebral hemorrhage or gastrointestinal bleed within the past 6 months, or major surgery within 30 days. A full list of inclusion and exclusion criteria is available in the published protocol.^3^

### Trial Procedures

Written informed consent was provided before any study specific procedures. As soon as possible after randomization, a loading dose of ticagrelor 180 mg (2×90 mg tablets) or matching placebo was to be given, followed by ticagrelor 90 mg or matching placebo twice daily, for the remainder of the 30-day treatment period. In addition, and as part of clinical practice, patients received a loading dose of aspirin (recommended 300–325 mg aspirin) and were subsequently treated with a recommended aspirin dose of 75 to 100 mg once daily. After the 30 days of study treatment, patients were treated according to standard of care at the discretion of the investigator and followed for an additional 30 days with continued collection of end points and safety events.

### Outcomes

All efficacy and safety analyses were based on investigator-assessed events, since applying central adjudication of outcome events in stroke trials does not improve data quality or impact on the estimated treatment effect in blinded, randomized clinical outcome trials.^7–9^ Stroke events were classified by investigators as ischemic, hemorrhagic, or of undetermined cause; those of undetermined cause were analyzed as ischemic strokes. Bleeding events were classified by the investigator according to the GUSTO trial (Global Utilization of Streptokinase and Tissue-Type Plasminogen Activator for Occluded Coronary Arteries) bleeding definition as severe, moderate, or mild.^10^ The definitions of the prespecified end points and GUSTO bleeding classification for this study have been previously described.^4,11^ The original primary efficacy end point was the time from randomization to the first subsequent event of stroke or death. The original primary safety end point was the time from randomization to the first GUSTO severe bleeding event. Patients experiencing neither event type by visit 3 (Day 34) were censored. For the current benefit-risk analysis, we compared major ischemic events (composite of ischemic stroke and nonhemorrhagic deaths) to major hemorrhage (composite of ICH and fatal bleedings) (Table). These composites were selected because both represent irreversible harm and capture the expected main benefits and the most important possible risks expected for antiplatelet drugs, while avoiding double counting of events.^12^ The modified Rankin Scale (mRS)^13^ was used to classify levels of disability at day 30 to further restrict outcomes to those secondary events resulting in disability. In addition, a net clinical impact end point was defined as a composite of ischemic stroke, ICH, fatal bleeding, and death, which includes all risk-benefit components of the 2 outcome measures.

**Table.**
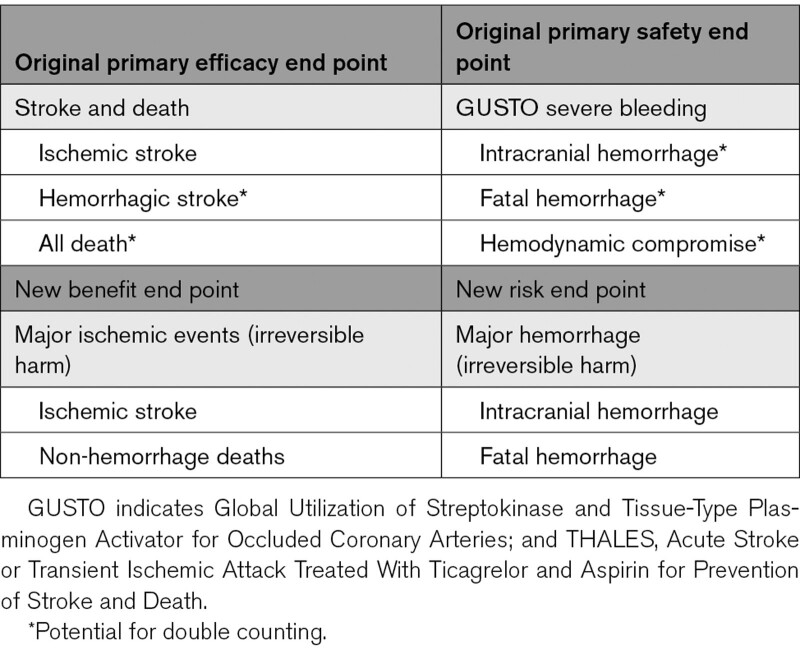
Original and New Risk-Benefit Outcome Assessments in THALES

### Statistical Analyses

Trial assumptions have been reported previously.^3,4^ All efficacy and safety analyses, including benefit-risk analyses, were based on the intention-to-treat principle using the full analysis set (including all randomized patients). Event rates for benefit and risk composites and their components were presented using Kaplan-Meier percentages at day 30. The absolute risk reductions (ARRs) in Kaplan-Meier percentages were calculated for the ticagrelor group versus the placebo group, along with 95% CIs. Number of events prevented/caused by treating 1000 patients with ticagrelor-aspirin was calculated as the ARR multiplied by 1000. Number needed to treat and number needed to harm were calculated as 1 divided by the ARR. Benefit and risk were analyzed in multiple models, including the original primary efficacy/safety end points, the new definitions, a more inclusive risk composite (GUSTO Severe/moderate bleeding events), and a more restrictive benefit composite (impact on disability).

ARRs and CIs were presented for subgroups with at least 5 events. The *P* value for interaction was calculated using the Cox proportional hazards model with treatment, subgroup, and their interaction as explanatory variables.

## Results

A summary of patient disposition is provided in the Data Supplement.

**Figure 1.**
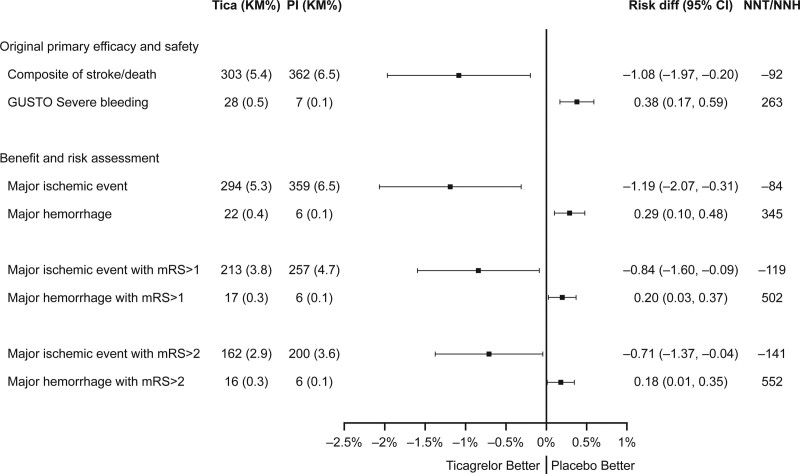
**Absolute risk differences between treatment with ticagrelor-aspirin and aspirin alone for the original outcome measures and for various disentangled measures of irreversible harm from ischemia and hemorrhage.** Bars indicate 95% CI. GUSTO indicates Global Utilization of Streptokinase and Tissue-Type Plasminogen Activator for Occluded Coronary Arteries; mRS, modified Rankin Scale; NNH, number needed to harm; and NNT, number needed to treat.

New disentangled composite measures of permanent injury from ischemia and hemorrhage were constructed from subcomponents of the original primary efficacy and safety outcomes of the trial (Table). The observed ARR for the ischemic benefit composite, major ischemia, with ticagrelor-aspirin at 30 days was 1.19% (95% CI, 0.31%–2.07%) and the increase of the bleeding risk composite, major hemorrhage, was 0.29% (95% CI, 0.10%–0.48%; Figure 1). The benefit was driven by a reduction of ischemic strokes (276 patients in the ticagrelor-aspirin group and 345 patients in the aspirin group). There were 19 deaths in each group when fatal bleeding and fatal ischemic stroke was excluded. The greater risk of hemorrhage in the ticagrelor-aspirin group was driven both by nonfatal ICH (11 versus 4) and fatal bleeding (11 versus 2). Transforming the absolute risk differences into patient numbers, treating 1000 patients with ticagrelor-aspirin for 30 days instead of aspirin alone is estimated to result in a reduction of 12 major ischemic events (composite of ischemic stroke and nonhemorrhagic death) and an increase of 3 major hemorrhages (composite of ICH and fatal bleeding).

To assure balance in the impact of events, additional analyses were performed restricting events to those producing any disability (mRS score >1) and moderate-to-severe disability (mRS score >2; Figure 1). The observed ARR for major ischemic events with mRS score >1 with ticagrelor-aspirin at 30 days was 0.84% (95% CI, 0.09%–1.60%), and the absolute risk increase of disabling hemorrhage was 0.20% (95% CI, 0.03%–0.37%). When only moderate-to-severe disabling events and deaths were counted (mRS score >2), the ARR for ischemic events was 0.71% (95% CI, 0.04%–1.37%), and the absolute risk increase of hemorrhage was 0.18% (95% CI, 0.01–0.35).

In comparison, the previously reported primary efficacy end point included both potential benefits and risks of antiplatelet therapy.^3^ Therefore, some events (ie, hemorrhagic strokes, fatal ICHs) are double-counted as both efficacy and safety events in the previous analysis (Table). Assessing the original primary efficacy end point, treatment with ticagrelor-aspirin resulted in a significant reduction in the rate of the composite of stroke and death with an ARR of 1.08% (95% CI, 0.20%–1.97%; Figure 1). Ticagrelor-aspirin was also associated with an increased risk of the primary safety end point, GUSTO severe bleeding (absolute risk increase of 0.38% [95% CI, 0.17%–0.59%]).

To assure that the impact of hemorrhage was not underestimated, a sensitivity analysis of the more inclusive risk composite, GUSTO moderate, or severe bleeding events, was performed, which includes all patients who have received a blood transfusion in addition to those with hemodynamic compromise, ICH, or fatal bleeding. The absolute risk increase of moderate-to-severe bleeding was 0.45% (95% CI, 0.21%–0.69%).

Number needed to treat for ticagrelor-aspirin ranged from 84 to 141 for the benefit end points: major ischemia (number needed to treat 84), original primary end point (92), any subsequent disabling stroke (119), and subsequent disabling stroke with moderate-to-severe disability (141) (Figure 1). Number needed to treat were consistently lower than the number needed to harm, which ranged from 221 to 552 for the risk end points: the major hemorrhage risk composite (number needed to harm 345), original primary safety end point (263), hemorrhagic risk composite with mRS score >1 (502), and hemorrhagic risk composite with mRS score >2 (552) and for the most inclusive risk end point, GUSTO moderate or severe bleeding (221).

**Figure 2.**
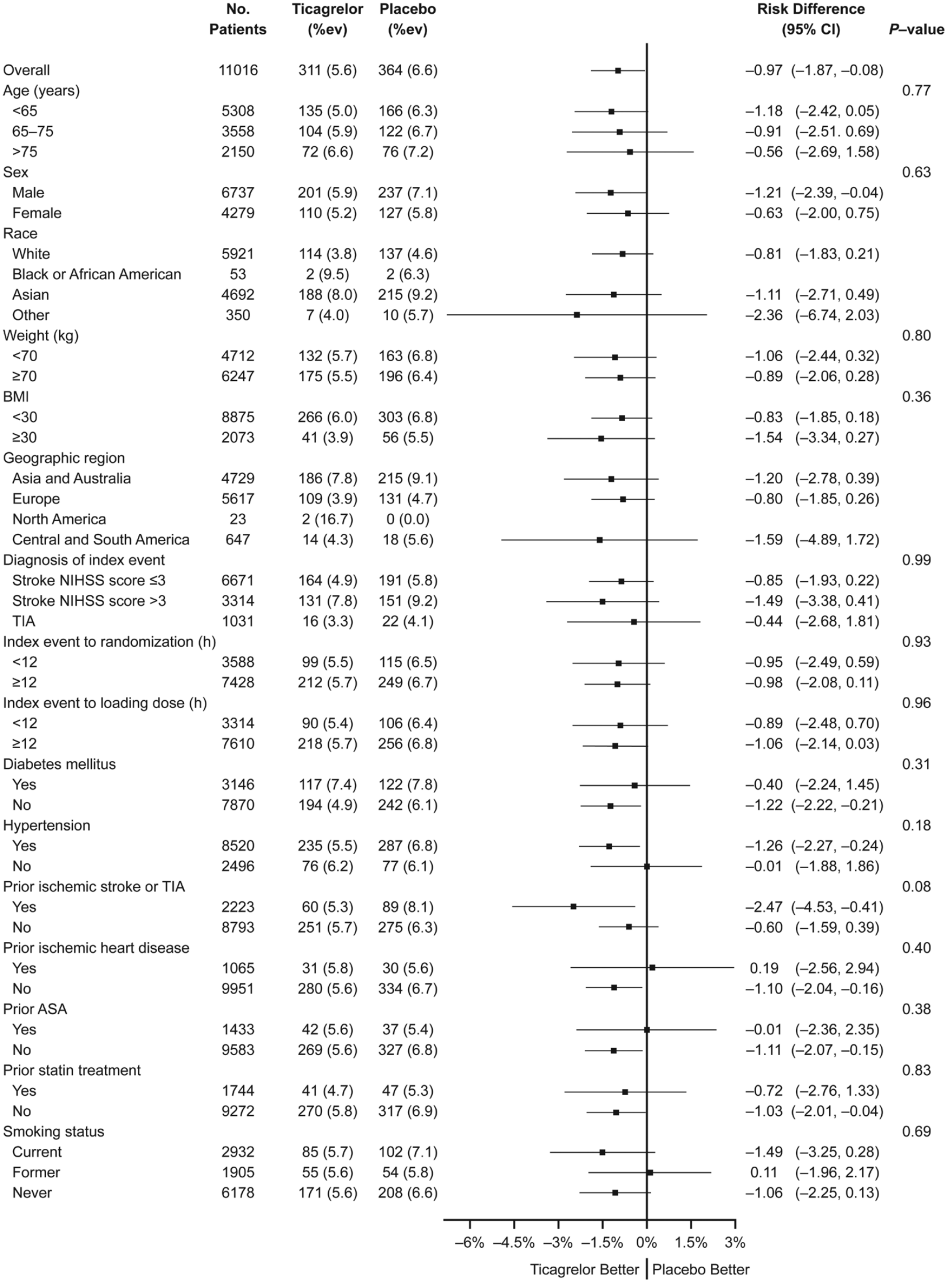
**Absolute risk differences between ticagrelor-aspirin and aspirin alone on net clinical impact (combining major ischemic events and major hemorrhage) in predefined subgroups.** Bars indicate 95% CI. No interaction was significant. BMI indicates body mass index; NIHSS, National Institutes of Health; and TIA, transient ischemic attack.

To complement the assessment of benefit and risk, we analyzed the impact of treatment on a net clinical impact end point, which was the composite of ischemic stroke, death, ICH, and fatal bleeding. Overall, 311 patients (5.6%) in the ticagrelor-aspirin group and 364 patients (6.6%) in the aspirin group had an event, corresponding to an ARR of 0.97% (95% CI, 0.08%–1.87%). Net clinical impact was explored in predefined subgroups corresponding to demographics, stroke risk factors, and characteristics of the index event (Figure 2). No significant interaction for any subgroup was observed.

**Figure 3.**
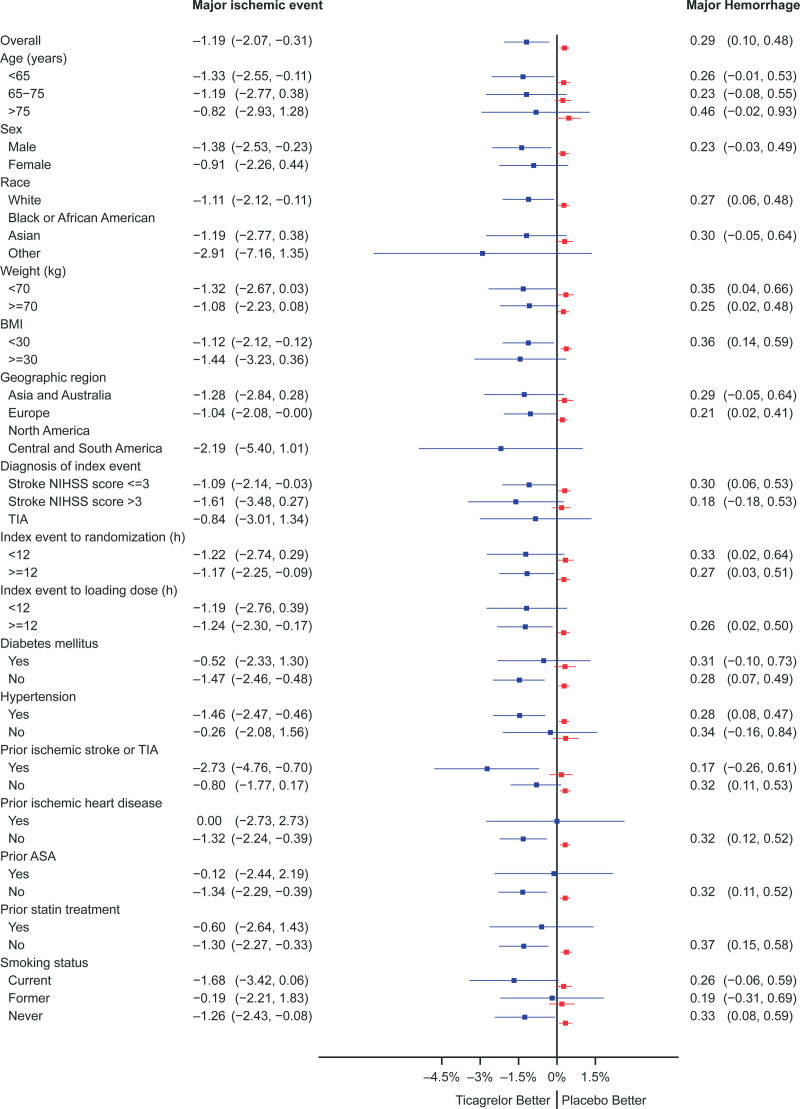
**Absolute risk differences between ticagrelor-aspirin and aspirin alone on major ischemic events (blue) and major hemorrhage (red) in predefined subgroups.** Bars indicate 95% CI. No interaction was significant. BMI indicates body mass index; NIHSS, National Institutes of Health; and TIA, transient ischemic attack.

Major ischemic events and major hemorrhage were analyzed in parallel by subgroups (Figure 3). The treatment effect in the ticagrelor-aspirin group compared with the aspirin group was consistent across all predefined subgroups. The point estimates for treatment effect were associated with large CIs that overlap, in some cases fully, with the CIs of other subgroups within the same category. The consistency of results in subgroups was illustrated by the lack of significant interaction. Some subgroups were small, including prior ischemic heart disease and no hypertension, which resulted in risk estimates with large CIs for ischemic benefit. For hemorrhagic risks, as there were few events overall, all subgroups should be interpreted with caution. No subgroup with different bleeding risk could be identified, and no subgroup had a distinctive risk-benefit profile.

A previous secondary analysis defined an atherosclerotic subgroup as those with ipsilateral cervical or intracranial arterial stenosis ≥30%.^14^ While the net clinical impact was numerically greater in those with atherosclerosis (ARR, 2.76% [95% CI, 0.38%–5.13%]) compared with those without (0.40% [95% CI, 0.53%–1.33%]), the interaction was not significant (*P*=0.26). Similarly, interactions were not significant for major ischemic events or major hemorrhage.

## Discussion

The primary results of the THALES trial raised confusion about how to balance risks and benefits of 30-day treatment with ticagrelor-aspirin among patients with acute mild-moderate ischemic stroke or high-risk TIA.^3^ Ignoring overlap in the primary efficacy and safety outcome measures and comparing relative risks rather than absolute risks obscured a direct comparison of benefits and harms. In the current analysis of end points that more clearly reflect benefits (reduction in major ischemic events) and harms (increase in major hemorrhage), we show that benefits of ticagrelor-aspirin outweigh risks over a range of disability levels and subgroups. When risks and benefits are combined into a net clinical impact end point, results continue to favor ticagrelor-aspirin over aspirin alone.

We sought to identify subgroups who might disproportionately benefit or be harmed by treatment. However, we found no such subgroups, over a range of predefined presenting conditions and demographics. While those with a history of prior stroke or TIA appeared to benefit more, the interaction term was not significant (*P*=0.08), and no other subgroups showed a trend toward a significant interaction. A previous analysis showed that those with ipsilateral atherosclerosis that could have accounted for the presenting stroke or TIA also appeared to benefit more and with a lower risk of hemorrhage, though interaction terms also were not significant,^14^ and a reanalysis with these new outcomes did not change this finding. Major hemorrhage was similar among the subgroups with no interactions apparent.

Benefits exceeded harms also when other definition of end points were used. When events were counted only if they resulted in any disability or moderate-severe disability, rates decreased but the relative benefit of ticagrelor-aspirin persisted. A broader inclusion of hemorrhages that encompassed moderate bleeds also did not tip the scales towards harm.

The original outcomes of the THALES trial with overlapping components of risk and benefit were chosen with encouragement from regulatory authorities but ultimately led to confusion. While the results of this reanalysis are consistent with the findings of the primary publication of the trial, the disentangled measures of benefit and risk are conceptually clearer and allow more direct comparison with prior trials. Furthermore, they clarify that no group receives disproportionate risk or benefit. The focus on absolute benefits avoids the common misconception that a high relative risk for a very rare event is more important than a smaller relative risk for a common event. Absolute risk differences can be weighted directly and better convey an individual’s risk of treatment. Based on this, we recommend that future trials utilize nonoverlapping outcomes for benefit and risk, and that absolute benefits are used to convey findings.^15^

This analysis has several limitations. First, while it is based on data from a randomized trial, it is a secondary analysis derived from new end point definitions. We attempted to evaluate the potential for bias in selecting the end points by evaluating several reasonable alternative end point definitions in sensitivity analyses, and these demonstrated the robustness of the findings. Second, there were very few major hemorrhages so it was difficult to identify predictors of hemorrhage. Third, we limited analysis of subgroups to predefined presenting characteristics and demographics. Other subgroup definitions and larger sample sizes might have revealed a population in whom risk would not be justified.

## Conclusions

In conclusion, this analysis from the THALES trial suggests that the benefits of 30-day treatment with ticagrelor-aspirin outweigh the risks. In treating 1000 patients with acute mild-moderate ischemic stroke or TIA, 12 major ischemic events would be expected to be avoided and 3 major hemorrhages would be produced compared with aspirin alone. The net benefits accrued across a spectrum of demographic and presenting characteristics.

## Acknowledgements

Editorial support (formatting and styling) for this article, under the direction of the authors, was provided by Lucy Helas, BSc, of Ashfield MedComms, an Ashfield Health company; this support was funded by AstraZeneca in accordance with Good Publication Practice (GPP3) guidelines (http://www.ismpp.org/gpp3).

## Sources of Funding

This study was supported by AstraZeneca.

## Disclosures

Dr S. Claiborne Johnston has received institutional research support from AstraZeneca and drug/placebo from Sanofi for a National Institutes of Health (NIH)-sponsored trial. Dr Pierre Amarenco reports receipt of research grant support from Pfizer, Sanofi, Bristol-Myers-Squibb, Merck, AstraZeneca, Boston Scientific, and from the French government, and consulting fees from Pfizer, BMS, Merck, Boehringer Ingelheim, AstraZeneca, Bayer, Daiichi Sankyo, Edwards, Boston Scientific, Kowa, GSK, Fibrogen, Amgen, Shing Poon, Gilead, and lecture fees from Bayer, St-Jude Medical, Amgen, Pfizer, Sanofi. Dr Scott Evans is a statistical consultant to AstraZeneca. Dr Maria Aunes, Marianne Jahreskog and Dr Sven Nylander are employees of AstraZeneca and hold stocks/shares in AstraZeneca. Drs Hans Denison, Anders Himmelmann, Mikael Knutsson, and Per Ladenvall are employees of AstraZeneca. Dr Stefan James has received institutional research grants from Astra Zeneca, The Medicines Company, Novartis, Bayer, and Jansen. He has also consulted for Medtronic. Dr Carlos Molina has received honoraria for participation in clinical trials, contribution to advisory boards, or oral presentations from AstraZeneca, Boehringer Ingelheim, Daiichi Sankyo, Bristol-Myers-Squibb, Covidien, Cerevast, and Brainsgate. Dr Joachim Röther has received honoraria from Astra Zeneca for oral presentations. Dr Yongjun Wang has received research grants from Sanofi, AstraZeneca and Amgen, and honoraria for participation to advisory board from Sanofi.

## Supplemental Materials

CONSORT diagram

## Supplementary Material


