# Supplementary material for: Ischemic Benefit and Hemorrhage Risk of Ticagrelor-Aspirin Versus Aspirin in Patients With Acute Ischemic Stroke or Transient Ischemic Attack
Source: Stroke. 2021 Sep 30;52(11):3482–9. doi: 10.1161/STROKEAHA.121.035555 (PMC8547576; doi:10.1161/STROKEAHA.121.035555)
Supplement: Supplementary file 2 [file str-52-3482-s002.pdf]

American Stroke Association.
A division of the
American Heart Association.

Acknowledgment Permission Form

Journal Stroke
Manuscript Number STROKE/2021/035555R1
First Author S. Claiborne Johnston
Title of Work mic Benefit And Hemorrhage Risk Of Ticagrelor-Aspirin Versus Aspirin In Patients With Acute Ischemic Stroke Or Transient Ischemic Attack

Authors must provide written permission/approval from all individuals mentioned by name in the Acknowledgments section of a submitted manuscript. By signing this form, any and all acknowledged persons therefore state that they have read and approved the mention of their names in the Acknowledgment section of the aforementioned paper.

	Name	Signature	Date
Name (1)	Lucy Helas	Lucy Helas Digitally signed by Lucy Helas Date: 2021.08.17 11:25:02 +01'00'	
Name (2)			
Name (3)			
Name (4)			
Name (5)			
Name (6)			
Name (7)			
Name (8)			
Name (9)			
Name (10)			
Name (11)			
Name (12)			
Name (13)			
Name (14)			
Name (15)			
Name (16)			
Name (17)			
Name (18)			
Name (19)			
Name (20)			
