# Supplementary figures and images for: Ischemic Benefit and Hemorrhage Risk of Ticagrelor-Aspirin Versus Aspirin in Patients With Acute Ischemic Stroke or Transient Ischemic Attack
Source: Stroke. 2021 Sep 30;52(11):3482–9. doi: 10.1161/STROKEAHA.121.035555 (PMC8547576; doi:10.1161/STROKEAHA.121.035555)

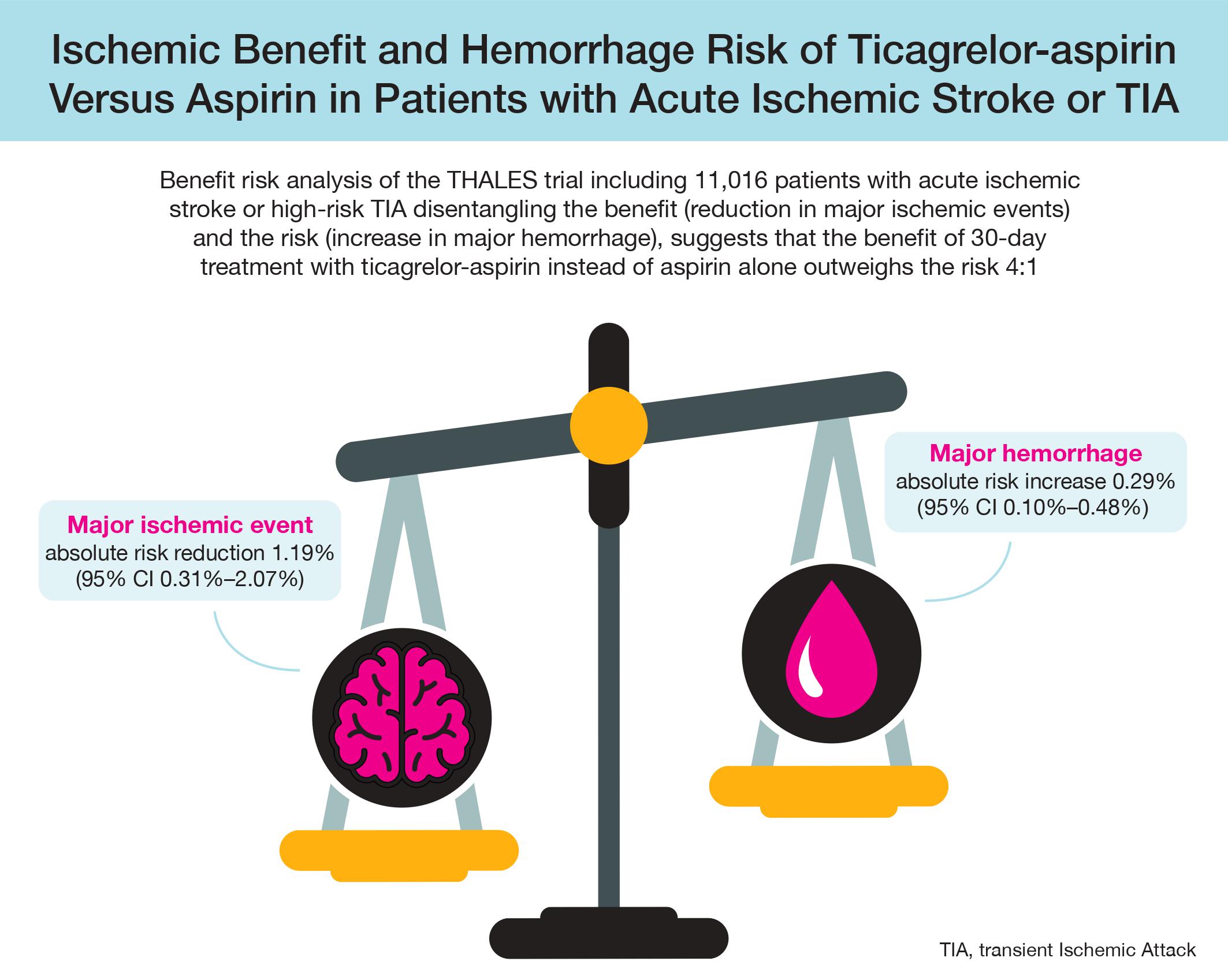

Supplement: Supplementary file 3 [file str-52-3482-s003.jpg]
